# Supplementary material for: Prediction of viral symptoms using wearable technology and artificial intelligence: A pilot study in healthcare workers
Source: PLoS One. 2021 Oct 14;16(10):e0257997. doi: 10.1371/journal.pone.0257997 (PMC8516235; doi:10.1371/journal.pone.0257997)
Supplement: S1 Table — Data dictionary of each data element used in the model. (PDF) [file pone.0257997.s001.pdf]

S1 Table. Data dictionary: data dictionary of each data element used in the model

Dictionary ID	Description
onset	The onset of disease. Target variable
Sleep duration	Duration of sleep
Sleep Score	Sleep Score computed by OURA Ring algorithm
Score Efficiency	Sleep Efficiency
Onset Latency	Sleep latency, the time before falling asleep
HR Lowest	Lowest heart rate during the night
HR	Average Heart Rate during night
HR base	HR variation from current participant average
HRV	Heart rate variability in ms
HRV base	Heart rate variability variation from current participant average
Temperature Delta	Variation of finger skin temperature from the current participant average
Temperature	Self-reported core temperature
Breath Average	Average breathing rate during the night
REM	Amount of REM sleep during the night
Light	Amount of Light sleep during the night
Deep	Amount of Deep sleep during the night
TLX Stress Score	TKX stress score
AM Readiness	Readiness score computed from morning questionnaires
MedianResponseTime AM	PVT morning Score

MedianResponseTime PM	PVT evening Score
Age	Age of the participant
**Engineered variables**	
E1 = REM / Light	Engineered variable*, Ratio of REM vs. Light sleep during the night of participant
E4 = HR / HRV	Engineered variable*, Ratio of HR vs. HRV during the night of participant
E5 = [HR] - [HRV]	Engineered variable*, HR minus HRV during the night of participant
E1 t1	Engineered variable*: E1 1 day ago
E1 t2	Engineered variable*: E1 2 days ago
E4 t1	Engineered variable*: E4 1 day ago
E4 t2	Engineered variable*: E4 2 days ago
E5 t1	Engineered variable*: E5 1 day ago
E5 t2	Engineered variable*: E5 2 days ago
HR delta	Engineered variable*: HR - HR base. Deviation of Heart Rate from the baseline level
HR delta t1	Engineered variable*: HR delta one day ago
HR delta t2	Engineered variable*: HR delta two days ago
HRV delta	Engineered variable*: HRV - HRV base. Deviation of Heart Rate Variability from the baseline level
HRV delta t1	Engineered variable*: HRV delta one day ago
HRV delta t2	Engineered variable*: HR delta two days ago

*Notation for engineered variables:

for any variable X

[X] - z-score normalization: [X] = (X - mean(X)) / std(X)

X base - time-average value for current participant

X delta = X - X base

X t1 - the value of X 1 day ago

X t2 - the value of X 2 days ago
