# Supplementary material for: Prediction of viral symptoms using wearable technology and artificial intelligence: A pilot study in healthcare workers
Source: PLoS One. 2021 Oct 14;16(10):e0257997. doi: 10.1371/journal.pone.0257997 (PMC8516235; doi:10.1371/journal.pone.0257997)
Supplement: S2 Table — The following table reports the probabilistic weights for each rule of the symptom onset forecasting model. (PDF) [file pone.0257997.s002.pdf]

S.7 Table: The following table reports the probabilistic weights for each rule of the symptom onset forecasting model.

**Rule**			**Classification**	**Weight**
IF	the score of labeling model for the day >= 0.5	then	suspicious	0.97
IF	the score of the labeling model for the day between 0.2 and 0.5	then	suspicious	0.95
IF	HRV between 30 and 43, and the score of the labeling model for the day < 0.2	then	suspicious	0.913
IF	Average breathing rate <= 14.5 and morning PVT median response time > 365	then	suspicious	0.895
IF	Age between 27 and 33, and the score of the labeling model for the day < 0.2	then	suspicious	0.868
IF	Sex = Female and score of labeling model for the day >= 0.5	then	suspicious	0.837
IF	Sleep onset latency > 0.0417 and REM sleep duration > 1.62	then	suspicious	0.83
IF	Age between 27 and 33 and score of labeling model for the day before < 0.2	then	suspicious	0.777
IF	Average breathing rate <= 14.5 and HRV > 43	then	suspicious	0.771
IF	HR delta <= -1.47 and Light sleep duration <= 3.42	then	suspicious	0.752
IF	Age > 46 and Sex = Female	then	suspicious	0.748
IF	morning PVT median response time between 322 and 365, and the score of the labeling model for the day < 0.2	then	suspicious	0.732
IF	Onset Latency between 0.00333 and 0.0417, and sleep score <= 72	then	suspicious	0.731
IF	Onset Latency between 0.00333 and 0.0417, and HRV delta t1 <= -4.11	then	suspicious	0.729
IF	AM readiness score <= 5.28 and Sex = Female	then	suspicious	0.72
IF	HR delta between -1.47 and 1.38 and sex = Male	then	suspicious	0.699
IF	E1 <= 0.274 and HRV baseline between 30.1 and 43.5	then	suspicious	0.693
IF	Evening PVT median response time between 326 and 375 and sex = Male	then	suspicious	0.635
IF	Onset Latency between 0.00333 and 0.0417 and score of labeling model for the day between 0.2 and 0.5	then	suspicious	0.581
	A priori	then	suspicious	0.116
IF	HR Lowest between 55 and 61 and TLX stress score between 88 and 163	then	not suspicious	0.873
IF	Age between 27 and 33 and sex = Female	then	not suspicious	0.863
IF	Light > 4.31 and evening PVT median response time > 375	then	not suspicious	0.856
IF	HRV between 30 and 43 and score of labeling model for the day before< 0.2	then	not suspicious	0.842
IF	HR delta t1 between -1.45 and 1.35 and REM > 1.62	then	not suspicious	0.829

IF	Sleep Score > 82 and HRV delta > 2	then	not suspicious	0.825
IF	Evening PVT median response time between 326 and 375 and sleep Score <= 72	then	not suspicious	0.808
IF	Morning PVT median response time between 322 and 365 and score of labeling model for the day before < 0.2	then	not suspicious	0.801
IF	E4 t2 between 1.44 and 2.32 and Score Efficiency between 83 and 96	then	not suspicious	0.792
IF	E1 t2 <= 0.27 and Light <= 3.42	then	not suspicious	0.779
IF	HR Lowest > 61 and HRV between 30 and 43	then	not suspicious	0.769
IF	E5 t1 between -0.753 and 0.724 and HR <= 62	then	not suspicious	0.766
IF	E1 <= 0.274 and HR delta between -1.47 and 1.38	then	not suspicious	0.765
IF	Onset Latency > 0.0417 and HRV delta t1 > 1.99	then	not suspicious	0.76
IF	E5 > 0.758 and HRV delta t1 <= -4.11	then	not suspicious	0.752
IF	Breath Average <= 14.5 and Sex = Female	then	not suspicious	0.741
IF	E5 t2 between -0.74 and 0.779 and temperature Delta between -0.1 and 0.08	then	not suspicious	0.739
IF	HR delta t2 between -1.45 and 1.43 and temperature <= 97.6	then	not suspicious	0.734
IF	Duration Integer HR <= 5.8 and E1 <= 0.274	then	not suspicious	0.731
IF	E4 t1 between 1.45 and 2.3 andE5 t1 between -0.753 and 0.724	then	not suspicious	0.719
IF	TLX Stress Score > 163 and HRV between 30 and 43	then	not suspicious	0.681
IF	Duration of light sleep period between 3.42 and 4.31 and sex = Male	then	not suspicious	0.671
IF	Age between 33 and 37.5 and TLX Stress Score <= 88	then	not suspicious	0.66
IF	Sex = Male and TLX Stress Score > 163	then	not suspicious	0.636
IF	Age in between 27 and 33	then	not suspicious	0.61
