# Supplementary material for: Prediction of viral symptoms using wearable technology and artificial intelligence: A pilot study in healthcare workers
Source: PLoS One. 2021 Oct 14;16(10):e0257997. doi: 10.1371/journal.pone.0257997 (PMC8516235; doi:10.1371/journal.pone.0257997)
Supplement: S1 File — List of questions asked to the participants. (PDF) [file pone.0257997.s003.pdf]

S2. List of Questions. List of questions asked to the participants.

Demographics:

1) Current Job Title and Department
2) Age:
3) Sex:
4) Home Zipcode
5) Work Zipcode
6) Height
7) Weight
8) Do you have Diabetes
9) Do you have High Blood Pressure (Hypertension)
10) Do you have a history of heart disease
11) Do you have a history of chronic lung disease (e.g., COPD, Asthma)
12) Does your current or past health history put you at risk of COVID-19
13) Household: number of people and age

Daily AM

1) Do you feel like you are getting sick (Y/N)
2) Do you have Fever (Y/N)
3) Do you have a Cough (Y/N)
4) Do you have Shortness of Breath (Y/N)
5) Coughing up Sputum or thick phlegm from the lungs (Y/N)
6) Bone or joint pain(Y/N)
7) Sore throat (Y/N)
8) Headache (Y/N)
9) Chills (Y/N)
10) Nausea or vomiting (Y/N)
11) Stuffy nose (Y/N)
12) Diarrhea (Y/N)
13) Coughing up blood (Y/N)
14) Swollen eyes (Y/N)

15) Has your sense of smell changed (Y/N)

16) Has your sense of taste changes (Y/N)

17) Have you been tested for COVID-19 (Y/N)

18) Are you Positive for COVID-19 (Y/N)

19) Are you positive for nonCOVID-19 illness (Y/N)

20) Rate your intellectual wellness (Memory, Attention, Focus) (0-100)

21) Rate your physical wellness (Health, Strength) (0-100)

22) Rate your emotional wellness (Feelings, Outlook on Life) (0-100

23) Did you have a hard time falling asleep (0-100)

24) Did you have a hard time getting out of bed (0-100)

25) How restorative was your sleep last night? (0-100)

26) Which one of these best describes how you feel right now?

   a. Extremely alert
   b. Very alert
   c. Alert
   d. Rather alert
   e. Neither alert nor sleepy
   f. Some signs of sleepiness
   g. Sleepy, but no effort to keep awake
   h. Sleepy, but some effort to keep awake
   i. Very sleepy, great effort to keep awake, fighting sleep
   j. Extremely sleepy, can't keep awake

<u>Short Recovery Scale</u>

27) Physical Performance Capability (e.g., strong, physically capable, energetic, full of power) (0-100)

28) Mental Performance Capability (e.g., attentive, receptive, mentally alert, concentrated) (0-100)

29) Emotional Balance (e.g., pleased, stable, in a good mood, having everything under control) (0-100)

30) Overall Recovery (e.g., recovered, rested, muscle relaxation, physically relaxed) (0-100)

<u>Short stress scale</u>

31) Muscular Stress (e.g., muscle exhaustion, muscle fatigue, muscle soreness, muscle stiffness) (0-100)

32) Lack of Activation (e.g., unmotivated, sluggish, unenthusiastic, lacking energy) (0-100)

33) Negative Emotional State (e.g., feeling down, stressed, annoyed, short-tempered) (0-100)

34) Overall Stress (e.g., tired, worn-out, overloaded, physically exhausted) (0-100)

Daily

Temperature: (#)

Daily PM

1) In the last 24 hours:

Have you encounter anyone who is Positive with COVID-19 (Y/N)

Have you encounter anyone who is being tested or concerned about having COVID-19 (Y/N)

Have you encountered anyone who has flu symptoms? (Y/N)

Number of people you interacted with at Work (i.e., Colleagues, patients, public areas) (#)

Number of people you interacted with outside Work (i.e., Family, friends, public settings) (#)

How many times did you wash your hands (#)

2) How many meals did you eat today (breakfast, lunch, dinner, snacks)?
3) What type of food did you eat today
   a. Pre-cooked foods
   b. Fresh foods
   c. Frozen foods
   d. Canned foods
   e. Cafeteria
   f. Restaurant
2) What shift did you work today (multiple select)
   a. Did not work
   b. Day
   c. Night
   d. 8 hr
   e. 12 hr

4) Did you exercise today longer than 30 min? (Y/N)

5) Do you take your time to rest and relax during that day?

6) Which one of these best describes how you feel right now?

   a. Extremely alert

   b. Very alert

   c. Alert

   d. Rather alert

   e. Neither alert nor sleepy

   f. Some signs of sleepiness

g. Sleepy, but no effort to keep awake

h. Sleepy, but some effort to keep awake

i. Very sleepy, great effort to keep awake, fighting sleep

j. Extremely sleepy, can't keep awake

7) How Mentally demanding was your day (0-100)

8) How physically demanding was your day (0-100)

9) How hurried or rushed was the pace of your day (0-100)

10) How successful were you in the accomplishing your daily tasks (0-100)

11) How hard did you have to work on accomplish your level of performance (0-100)

12) How insecure, discourage, irritated, stressed, and annoyed were you today (0-100)
