# Supplementary material for: Prediction of viral symptoms using wearable technology and artificial intelligence: A pilot study in healthcare workers
Source: PLoS One. 2021 Oct 14;16(10):e0257997. doi: 10.1371/journal.pone.0257997 (PMC8516235; doi:10.1371/journal.pone.0257997)
Supplement: S2 File — Inclusion/exclusion criteria and description of data set. (PDF) [file pone.0257997.s004.pdf]

S3. Data Set & Inclusion/Exclusion criteria

S. 3.1 Fig.: Inclusion/exclusion criteria for both models, with and without cognitive assessment.

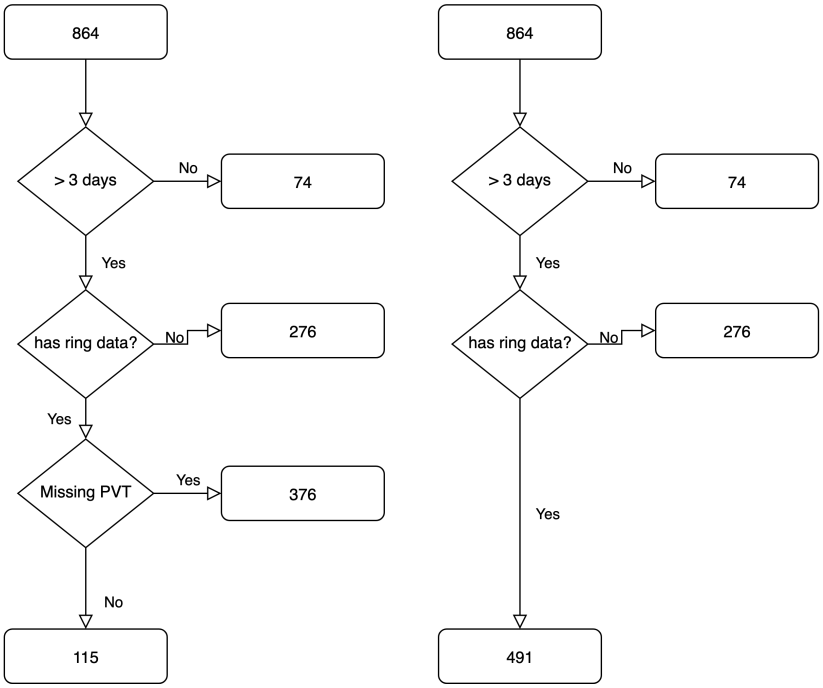

S.3.2 Fig. Dataset. The figure illustrates the distribution of the key elements used in a) the labeling model, b) the prediction model.

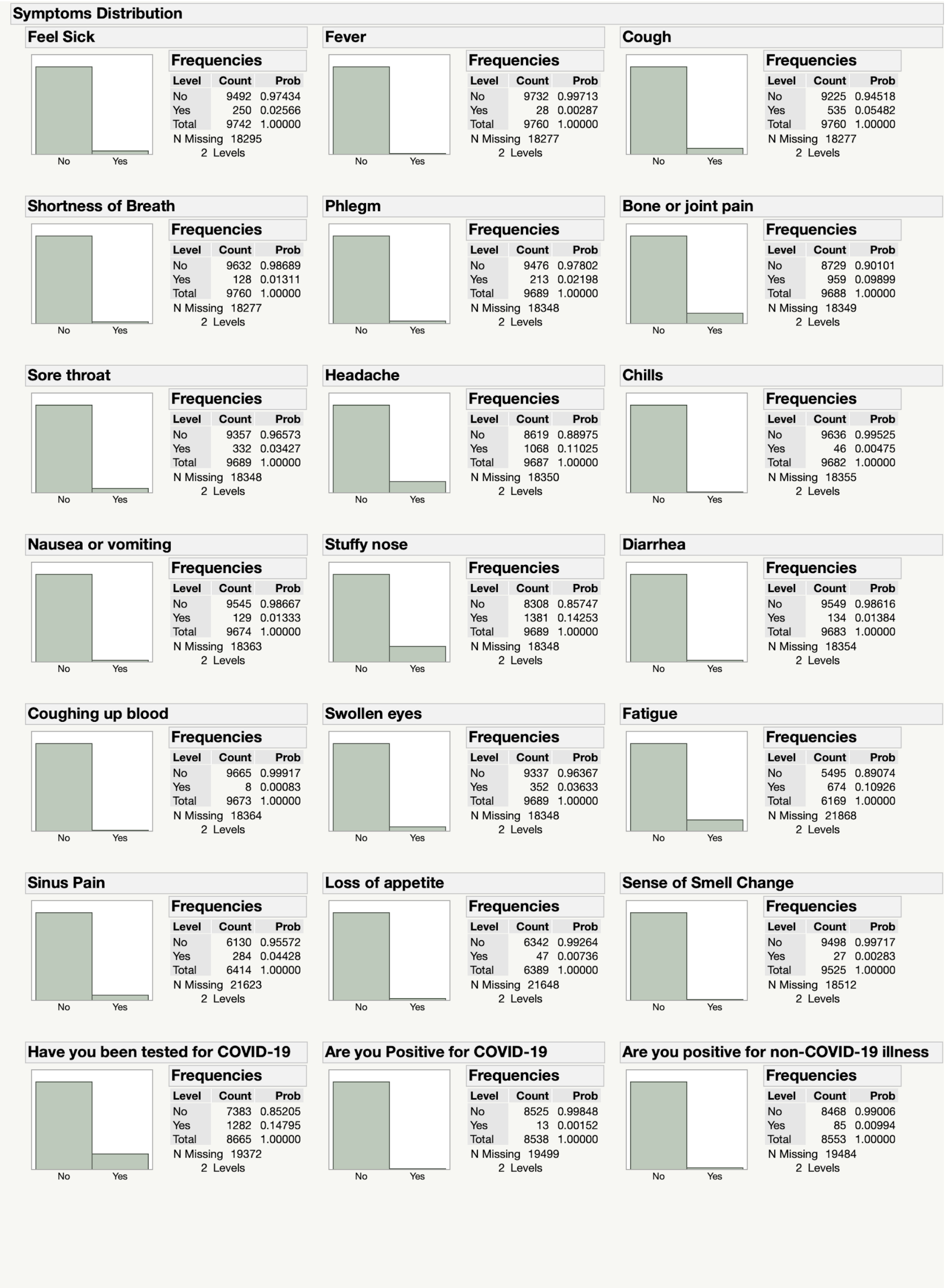

Symptoms Distribution

Feel Sick — Frequencies

Level	Count	Prob
No	9492	0.97434
Yes	250	0.02566
Total	9742	1.00000

N Missing 18295
2 Levels

Fever — Frequencies

Level	Count	Prob
No	9732	0.99713
Yes	28	0.00287
Total	9760	1.00000

N Missing 18277
2 Levels

Cough — Frequencies

Level	Count	Prob
No	9225	0.94518
Yes	535	0.05482
Total	9760	1.00000

N Missing 18277
2 Levels

Shortness of Breath — Frequencies

Level	Count	Prob
No	9632	0.98689
Yes	128	0.01311
Total	9760	1.00000

N Missing 18277
2 Levels

Phlegm — Frequencies

Level	Count	Prob
No	9476	0.97802
Yes	213	0.02198
Total	9689	1.00000

N Missing 18348
2 Levels

Bone or joint pain — Frequencies

Level	Count	Prob
No	8729	0.90101
Yes	959	0.09899
Total	9688	1.00000

N Missing 18349
2 Levels

Sore throat — Frequencies

Level	Count	Prob
No	9357	0.96573
Yes	332	0.03427
Total	9689	1.00000

N Missing 18348
2 Levels

Headache — Frequencies

Level	Count	Prob
No	8619	0.88975
Yes	1068	0.11025
Total	9687	1.00000

N Missing 18350
2 Levels

Chills — Frequencies

Level	Count	Prob
No	9636	0.99525
Yes	46	0.00475
Total	9682	1.00000

N Missing 18355
2 Levels

Nausea or vomiting — Frequencies

Level	Count	Prob
No	9545	0.98667
Yes	129	0.01333
Total	9674	1.00000

N Missing 18363
2 Levels

Stuffy nose — Frequencies

Level	Count	Prob
No	8308	0.85747
Yes	1381	0.14253
Total	9689	1.00000

N Missing 18348
2 Levels

Diarrhea — Frequencies

Level	Count	Prob
No	9549	0.98616
Yes	134	0.01384
Total	9683	1.00000

N Missing 18354
2 Levels

Coughing up blood — Frequencies

Level	Count	Prob
No	9665	0.99917
Yes	8	0.00083
Total	9673	1.00000

N Missing 18364
2 Levels

Swollen eyes — Frequencies

Level	Count	Prob
No	9337	0.96367
Yes	352	0.03633
Total	9689	1.00000

N Missing 18348
2 Levels

Fatigue — Frequencies

Level	Count	Prob
No	5495	0.89074
Yes	674	0.10926
Total	6169	1.00000

N Missing 21868
2 Levels

Sinus Pain — Frequencies

Level	Count	Prob
No	6130	0.95572
Yes	284	0.04428
Total	6414	1.00000

N Missing 21623
2 Levels

Loss of appetite — Frequencies

Level	Count	Prob
No	6342	0.99264
Yes	47	0.00736
Total	6389	1.00000

N Missing 21648
2 Levels

Sense of Smell Change — Frequencies

Level	Count	Prob
No	9498	0.99717
Yes	27	0.00283
Total	9525	1.00000

N Missing 18512
2 Levels

Have you been tested for COVID-19 — Frequencies

Level	Count	Prob
No	7383	0.85205
Yes	1282	0.14795
Total	8665	1.00000

N Missing 19372
2 Levels

Are you Positive for COVID-19 — Frequencies

Level	Count	Prob
No	8525	0.99848
Yes	13	0.00152
Total	8538	1.00000

N Missing 19499
2 Levels

Are you positive for non-COVID-19 illness — Frequencies

Level	Count	Prob
No	8468	0.99006
Yes	85	0.00994
Total	8553	1.00000

N Missing 19484
2 Levels

Distributions

Sleep Duration

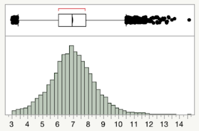

Summary Statistics	
Mean	6.9343047
Std Dev	1.443121
Std Err Mean	0.0118336
Upper 95% Mean	6.9575001
Lower 95% Mean	6.9111094
N	14872
Median	6.92
Range	11.64

Sleep Score

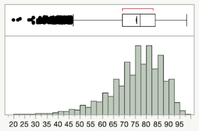

Summary Statistics	
Mean	75.475054
Std Dev	11.35598
Std Err Mean	0.0931194
Upper 95% Mean	75.657579
Lower 95% Mean	75.292528
N	14872
Median	77
Range	78

Score Efficiency

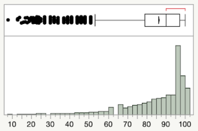

Summary Statistics	
Mean	86.228954
Std Dev	14.552462
Std Err Mean	0.1193306
Upper 95% Mean	86.462856
Lower 95% Mean	85.995051
N	14872
Median	90
Range	92

Onset Latency

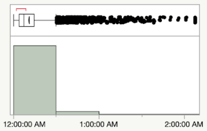

Summary Statistics	
Mean	653.96651
Std Dev	696.54597
Std Err Mean	5.7116961
Upper 95% Mean	665.16214
Lower 95% Mean	642.77088
N	14872
Median	450
Range	7650

HR Lowest

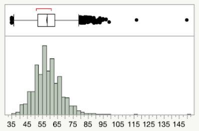

Summary Statistics	
Mean	58.03783
Std Dev	8.5439239
Std Err Mean	0.0700981
Upper 95% Mean	58.175231
Lower 95% Mean	57.900429
N	14856
Median	58
Range	115

HR

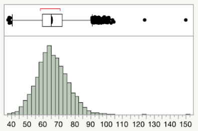

Summary Statistics	
Mean	65.683475
Std Dev	9.5096961
Std Err Mean	0.0780218
Upper 95% Mean	65.836407
Lower 95% Mean	65.530542
N	14856
Median	65.1
Range	111.9

HR_base

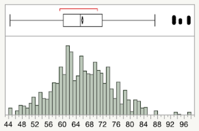

Summary Statistics	
Mean	65.853224
Std Dev	8.4837355
Std Err Mean	0.0644374
Upper 95% Mean	65.979528
Lower 95% Mean	65.72692
N	17334
Median	65.217568
Range	52.949287

HRV

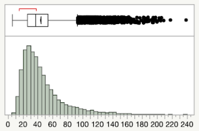

Summary Statistics	
Mean	44.04591
Std Dev	27.362938
Std Err Mean	0.2245052
Upper 95% Mean	44.485968
Lower 95% Mean	43.605853
N	14855
Median	37
Range	233

HRV_base

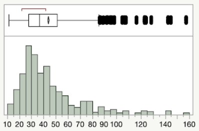

Summary Statistics	
Mean	43.817322
Std Dev	24.703689
Std Err Mean	0.1876344
Upper 95% Mean	44.185105
Lower 95% Mean	43.44954
N	17334
Median	36.724638
Range	146.39535

Temperature Delta

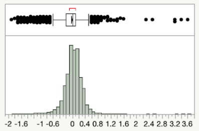

Summary Statistics	
Mean	-0.026247
Std Dev	0.2695224
Std Err Mean	0.0022159
Upper 95% Mean	-0.021904
Lower 95% Mean	-0.030591
N	14794
Median	0
Range	5.5

Temperature

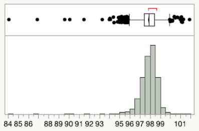

Summary Statistics	
Mean	97.848245
Std Dev	0.7687132
Std Err Mean	0.0087399
Upper 95% Mean	97.865377
Lower 95% Mean	97.831112
N	7736
Median	97.9
Range	17.8

Breath Average

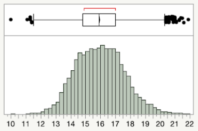

Summary Statistics	
Mean	15.919829
Std Dev	1.5746897
Std Err Mean	0.0129125
Upper 95% Mean	15.945139
Lower 95% Mean	15.894519
N	14872
Median	15.9
Range	11.9

REM

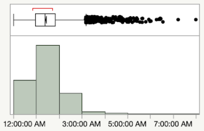

Summary Statistics	
Mean	5176.1377
Std Dev	2562.9308
Std Err Mean	21.016103
Upper 95% Mean	5217.3319
Lower 95% Mean	5134.9436
N	14872
Median	5010
Range	28800

Light

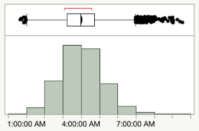

Summary Statistics	
Mean	14437.405
Std Dev	4081.0171
Std Err Mean	33.464453
Upper 95% Mean	14503
Lower 95% Mean	14371.811
N	14872
Median	14310
Range	31980

Deep

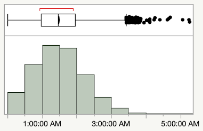

Summary Statistics	
Mean	5349.2469
Std Dev	2571.5369
Std Err Mean	21.086674
Upper 95% Mean	5390.5794
Lower 95% Mean	5307.9144
N	14872
Median	5250
Range	18960

TLX Stress Score

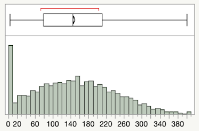

Summary Statistics	
Mean	146.6897
Std Dev	89.283976
Std Err Mean	0.9388955
Upper 95% Mean	148.53015
Lower 95% Mean	144.84926
N	9043
Median	145
Range	396

AM_Readiness

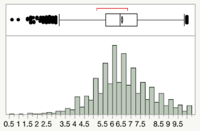

Summary Statistics	
Mean	6.5170909
Std Dev	1.4383607
Std Err Mean	0.0145728
Upper 95% Mean	6.5456567
Lower 95% Mean	6.4885252
N	9742
Median	6.4
Range	9.3333333

MedianResponseTime_AM

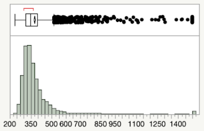

Summary Statistics	
Mean	376.67278
Std Dev	134.35218
Std Err Mean	1.6014748
Upper 95% Mean	379.81215
Lower 95% Mean	373.5334
N	7038
Median	346.5
Range	1262.5

MedianResponseTime_PM

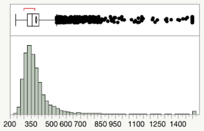

Summary Statistics	
Mean	386.29605
Std Dev	135.07806
Std Err Mean	1.541762
Upper 95% Mean	389.31833
Lower 95% Mean	383.27378
N	7676
Median	357.5
Range	1258.5

Age

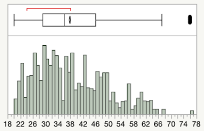

Summary Statistics	
Mean	37.715801
Std Dev	11.326706
Std Err Mean	0.0797556
Upper 95% Mean	37.872129
Lower 95% Mean	37.559474
N	20169
Median	36
Range	56

E1

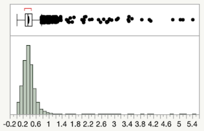

Summary Statistics	
Mean	0.3719478
Std Dev	0.2205676
Std Err Mean	0.0018087
Upper 95% Mean	0.375493
Lower 95% Mean	0.3684026
N	14872
Median	0.3504748
Range	5.4095238

E4

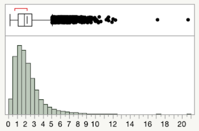

Summary Statistics	
Mean	2.1240533
Std Dev	1.4360376
Std Err Mean	0.0117823
Upper 95% Mean	2.147148
Lower 95% Mean	2.1009585
N	14855
Median	1.8055556
Range	20.495886

E5

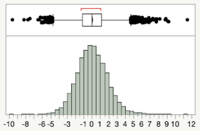

Summary Statistics	
Mean	0.0019769
Std Dev	1.7951073
Std Err Mean	0.0147328
Upper 95% Mean	0.0308551
Lower 95% Mean	-0.026901
N	14846
Median	-0.044599
Range	21.070165
