# Supplementary material for: Prediction of viral symptoms using wearable technology and artificial intelligence: A pilot study in healthcare workers
Source: PLoS One. 2021 Oct 14;16(10):e0257997. doi: 10.1371/journal.pone.0257997 (PMC8516235; doi:10.1371/journal.pone.0257997)
Supplement: S3 File — Detailed description of the Probabilistic Rule Engine. (PDF) [file pone.0257997.s005.pdf]

S4. Probabilistic Rule Engine

The probabilistic graphical network defined by the system of rules and corresponding weights postulates a probability distribution as

$$P(x) = \frac{1}{Z} exp(\sum_j \omega_j \ f_j(x))$$

where $x = (x_1, x_2, and., x_{n+1})$ is a set of binary variables (among which there are n input and one output variable), $f_j(x) \in \{1, 0\}$ is a boolean function corresponding to the jth logical formula, ω_j is the weight associated with the logical formula, Z is the normalization constant. In the current implementation, the relation between the rule's weight ω and confidence level ψ is given by $\psi = \frac{exp(\omega)}{1 + exp(\omega)}$.

The probability distribution is calibrated to the training set using a cross-entropy loss function. The calibration allows finding the set of weights ω_j which maximizes the likelihood of the observation. The model prediction s for every observation $r = (r_1, r_2, and., r_n)$ is computed as the conditional probability of the output variable y: $s = P(y = 1|r)$

The numerical implementation of the calibration routine is based on the Limited-memory Broyden–Fletcher–Goldfarb–Shanno algorithm (L-BFGS). We chose this method for fast convergence and efficient use of computational resources.
