# Supplementary material for: Prediction of viral symptoms using wearable technology and artificial intelligence: A pilot study in healthcare workers
Source: PLoS One. 2021 Oct 14;16(10):e0257997. doi: 10.1371/journal.pone.0257997 (PMC8516235; doi:10.1371/journal.pone.0257997)
Supplement: S4 File — Detailed description of the validation approach. (PDF) [file pone.0257997.s006.pdf]

S5 Validation Approach

K-fold cross-validation: The model performance was tested using 25% of the data reserved for testing. To avoid overfitting, we repeated the validation procedure by splitting the dataset four times into 75/25 ratios so all points can be used as training and testing but never at the same time. The results of the four validation runs are then averaged to get the final results. This procedure is commonly called K-fold cross-validation and is used to remove bias from an arbitrary selection of testing and training datasets.

Model stability: To study the model's stability, we randomly truncate the original dataset into smaller data of different sizes. The model is then trained and tested for each dataset size, and performances are reported as a function of the dataset size. Such a technique is used to measure the stability of the model and assess when additional data would not significantly affect the model's performance. From the learning curve presented in the table, we can infer that the model performance saturates after about 1000 data points and gains marginal performance after the number of data points is doubled or tripled (e.g., at the size 1100 points, the AUC is 0.76, and at 3000 points, the AUC is 0.79). This indicates that the model based on the currently available data is not expected to benefit noticeably from the further increase of the training data size within an order of magnitude
