# Supplementary material for: Prediction of viral symptoms using wearable technology and artificial intelligence: A pilot study in healthcare workers
Source: PLoS One. 2021 Oct 14;16(10):e0257997. doi: 10.1371/journal.pone.0257997 (PMC8516235; doi:10.1371/journal.pone.0257997)
Supplement: S5 File — Detailed description of the labeling model. (PDF) [file pone.0257997.s007.pdf]

S6. Labeling Model

The labeling model goal is to combine the symptoms self-reported by each participant every day into a binary decision of being suspicious (or not) of presenting symptoms consistent with a viral illness on the day of reported symptoms. This process tries to emulate the common diagnosis of viral-like illness and those more specific for SARS-Cov-2 (e.g., loss of taste and smell). To reach a model that can, with accuracy, combine inputs from participants into a binary decision, we suggest a rule-based approach. First, a panel of experts, with the help of the most recent literature review, provided a list of conditions that will make some more or less suspicious of presenting symptoms consistent with a viral illness (Table S.6.1). We then create synthetic datasets that would match these conditions and trained a probabilistic rule engine model, as explained in the previous section. The list of rules with their corresponding weight extracted by the algorithm is listed in Table S.6.2. Rules are associated through the equation in the section above. The model outputs a traditional probability, ranging from 0 to 1, of being suspicious of presenting symptoms consistent with a viral illness. A cutoff threshold (0.72) is then chosen to maximize the model performance on matching the experts' rules.
