# Supplementary material for: Prediction of viral symptoms using wearable technology and artificial intelligence: A pilot study in healthcare workers
Source: PLoS One. 2021 Oct 14;16(10):e0257997. doi: 10.1371/journal.pone.0257997 (PMC8516235; doi:10.1371/journal.pone.0257997)
Supplement: Attachment — Submitted filename: Response to Reviewers (1).docx [file pone.0257997.s008.docx]

# Response to Reviewers

First, we would like to thank the Reviewers for the detailed reading of our manuscript and all the valuable comments. Please find below a list of changes in response to their comments.

## Responses to the comments of the first Reviewer:

### 1. The Reviewer wrote:

“First off, the machine learning component is not clear. The model is described as a probabilistic graphical network acting on a combination of binary features. The exact operation or preprocessing applied to these features, or indeed what these features are, is not specified in the main paper. Further information is supplied in the supplementary material, however it would enhance reader understanding considerably to give some clearer examples on, for example, how measured heart rate is incorporated into the model. (Even in the supplementary material, the model description could be improved.) This whole section should be written in a clearer, fuller, way using appropriate terminology to allow easier replication. It would also be helpful to know why this model was chosen over other potential machine learning approaches.”

Our reply:

To address the reviewer’s comments, we rewrote the section about forecasting to clarify our approach. In particular, we incorporated some of the supplementary material into the main text to add more information about the underlying algorithm in this section and the relevant references to Markov Logic. We also added information about the data flow and the preprocessing to facilitate the replication of our approach.

### 2. The **Reviewer** wrote:

“The training and testing procedure of the algorithm is also unclear. The supplementary material goes some way to clarifying this, but the main text could be tightened up somewhat. As K-fold cross-validation is used, it would help to directly specify the value of K (=4 in this case). Specifying a 25% test set split is not quite enough because this could imply a single leave-one test set out evaluation. With the results being averaged over the K folds, then some additional measure of variance might be helpful. Following from this, the supplementary material provides a table of model weights - how are these arrived at from the K-folds?”

Our reply:

We made the appropriate changes to the main text; in particular, we specified the value of K=4:

“Model performance was tested with K-fold cross-validation using the reserved 25% portion of the data, i.e. K=4 in our case.”

As usual in supervised learning, the model weights in the final model are not found via averaging but instead obtained by using all available data to train the model. To clarify this in the main text, we added the following sentence:

“The model weights in the final model are obtained by using all available data to train the model.”

The differences in the AUCs of the different folds were too small to be of interest. Moreover, our samples are appropriately stratified, avoiding a single leave-one test set out evaluation.

### 3. **The Reviewer wrote:**

“Finally, the results presentation should be improved. The use of several complementary metrics makes sense; however the headline result in the abstract and discussion present misleading figures. The abstract reports a positive recall, or sensitivity, of 97%, when in fact it should be 79%. According to the tables, the negative recall, or specificity, should be around 83%; however, the discussion states that "almost all of the time (97%), individuals who will not develop viral-like illness symptoms". There may be other errors or confusions in the reporting of these results that I have not found, and I would recommend going through these in detail again.”

Our reply:

We agree with the Reviewer and modified our report of results to increase clarity. The numbers in the abstract refer to Table A, particularly to the column reporting results for the threshold chosen to be 0.1. For this case, the sensitivity is indeed 79%, and the 93% refer to the negative predictive value (TN/(TN+FN)). We made the following changes in the text to improve clarity:

“We describe our strategy using an AI model that can predict, with 82% accuracy (negative predictive value 97%, specificity 83%, sensitivity 79%, precision 34%)”

We also added the words “negative predictive value” and “precision” to the main text:

The model would detect 79% of individuals who will develop symptoms (i.e., sensitivity) and correctly predicts, almost all of the time (97%, negative predictive value), individuals who will not develop viral-like illness symptoms in the next three days. Conversely, the model precision is 34%. Remember that precision is defined as the ratio of true positives (TP) over positives (P). In other words, if the model flags someone to develop viral-like symptoms in the next three days, the model is correct 34% of the time.

### 4. **The Reviewer wrote:**

“Some specific presentation issues:

- Introduction, para 3: sentence structure on 'Outputs from wearable...health and disease'

- See also 'Besides, subject-reported ... COVID-19'”

Our reply:

We changed the sentences that the reviewer pointed out. The new version reads:

Outputs from wearable technology, including heart rate (HR), heart rate variability (HRV), respiration rate (RR), temperature, blood oxygenation, sleep, and other physiological assessments, are increasingly being explored in studies of health and disease [^10–12^](https://paperpile.com/c/5iiCIY/HTRIL+G9dzh+3Xwtg). Moreover, various subject-reported symptoms captured on mobile apps transform both surveillance and contact tracing management strategies for COVID-19 [^13–15^](https://paperpile.com/c/5iiCIY/oBQLe+u0DYN+d7rzv).

### 5. **The Reviewer wrote:**

“Forecasting Model: unclear statement 'where x is a set of binary variables (among which there are n input and one output variable)...' What does n refer to? This whole section needs expanding and clarification.”

Our reply:

We rewrote this section to address the Reviewer’s comments. We rephrased the paragraph as follows:

The joint distribution defined by the Markov network can be written as where denotes a set of *n+1* binary variables, out of which the first *n* are input variables, and *y* is the output variable. Here, is a Boolean function corresponding to the rule, is a factor associated with the corresponding rule, is the normalization constant.

### 6. The Reviewer wrote:

“Results: 867 -> 767 -> 115. Was it not possible to salvage some of the missing data? For example, include some of the 376 missing cognitive data that had some wearable data towards training the forecasting model?”

Our reply:

In principle, we agree with the Reviewer that some of this data could be attributed appropriately to train the forecasting model. We did some research in this direction, but the preliminary results based on naïve imputation techniques were not very encouraging such that more research is necessary in order to salvage the missing data. In order to capture this in the text, we added the following sentence in the discussion:

We plan to develop additional models to attribute the data efficiently to extend the forecasting model’s usability.

**Responses to the comments of the second Reviewer:**

## 1. The Reviewer wrote:

“1. The authors state that "The model is calibrated using an accepted method (cross-entropy loss function, see supplementary material) that finds the set of weights , which minimizes the prediction errors using the training model". After checking the supplementary material, I found the authors state that the cross-entropy function maximizes the maximum likelihood estimation (MLE). However, it is not clear to me what is the y in the process to calculate the conditional probability P(y=1|r), the y is not explained clearly in supplementary material (SM). Is it the subjects in the study who were eventually infected by COVID-19? Moreover the cross entropy loss function is Loss = -y*log(y). This term is then summed over all the possible classes in the dataset. How are the rules fit into this framework? If the authors are not using something like this how are weights calculated?

This calculation of weights is a very important detail in the machine learning (ML) model developed by the authors but it is a bit unclear. I would like the authors to explain it in greater detail and write about it in the main manuscript rather than SM.”

Our reply:

To address the reviewer’s concerns, we rewrote the section on the forecasting model and included more material from the supplementary material in the main text. This also includes relevant references in the main text.

## 2. The Reviewer wrote:

“2. The authors use the same line twice in the paper, once in abstract and once in the discussion.

"Conversely, the model correctly predicts as positive, 34% of the time, individuals who will develop viral-like illness symptoms in the next three days. "

Details about the implication of the statement in the discussion section are missing.

What does 34% signify in this case? Doesit mean that the accuracy for viral-likesymptoms is 34%?”

Our reply:

To clarify the text, we added the word “precision” to the sentence in the abstract. Precision is defined as the ratio TP/P = TP/(TP+FP) (where TP are the true positives and FP are the false positives). We also made the following changes in the discussion:

Conversely, the model precision is 34%. Remember that precision is defined as the ratio of true positives (TP) over positives (P). In other words, if the model flags someone to develop viral-like symptoms in the next three days, the model is correct 34% of the time.

## 3. The Reviewer wrote:

“3. Are the rules stated in the forecasting model created by experts after observing the data? If not, how were they created? The rules in Table 6.1 clearly state expert rule used in labelling model but there is no such mention of that in table 7.1. Assuming it was created by experts it would have been more useful to use some ML techniques that figure about the rules themselves like random forests and other decision tree based models.”

Our reply:

The rules used in the forecasting model are found via rule mining from our algorithm. The rewritten section on forecasting now contains more information to clarify this point. In particular:

To create the forecasting model, however, the rules were extracted from the available data via rule mining and the weights of the rules were determined by the calibration process.

Also, note that we were focusing on developing an interpretable rule-based model consisting of a relatively small number of rules in our approach. Random forest usually produces a large number of rules, which makes this approach much less interpretable. In our research, the probabilistic rules yielded the most promising results when developing an accurate yet transparent model.

## 4. The Reviewer wrote:

“4.Intuitively, the greater weight would imply greater influence of the rule in predicting the label. However, most of the weights presented in the study are fairly close. For example: Shortness of Breath, Coughing up blood, Sore throat, Chills, Phlegm, Diarrhea have almost the same weight ~0.82. No analysis of why this is happening has been presented, is it because of the nature of the data? some of the parameters presented in the example are distinctly different. Another thing that I found interesting was that persistence of symptoms had lesser weight than cough and fever. However, if the stable’s symptoms are persistent for 2-3 days, the person would be more worried. It would be interesting to analyze the weights in greater detail. It will make the study clearer and more accessible.”

Our reply:

The rules and weights of the labeling were dictated from the input of medical and epidemiologists experts. This is different from the forecasting model in which the rules are found via rule mining by our algorithm and the weights set through algorithm calibration. To clarify the origin of the rules and weights of the labeling model, we added the following sentences:

The rules and weights in this model were found from expert input and reviewed by our medical team. In particular, the weights associated with the rules were chosen to minimize the labeling error assessed by medical experts. We also fine-tuned some rule weights by fitting the model to a small synthetic data set containing a few most typical symptom combinations. The actual calculation of the labeling model’s output score happens by applying the probabilistic logic machinery, described in more detail in the next paragraph.

We agree with the Reviewer that the labeling model itself, together with the rules and weights, would warrant a broader discussion. In this paper, however, the labeling model is mainly used to define the output variable and thus set the forecasting model stage, which is in this paper’s focus. However, we plan to extend the discussion with our medical team and look into the weights more in-depth to publish insights focusing on the labeling model itself.

## 5. The Reviewer wrote:

“5.This is a minor concern: The very essence of ML lies in learning from the features and developing a mapping between labels (in this case the rules and labels). If the authors use some more advanced ML models in their future studies perhaps there will no need to draft the rules. One problem that I see that the designed rules may not be exhaustive and some of the important rules may be missing from the model in the current state.”

Our reply:

We assume that the Reviewer’s comments are in regard to the labeling model. The Reviewer is correct in the sense that rule-based systems are most powerful when there is a combination of expert input and rule-mining from the data. In the particular case of the labeling model in this study, the output variable needed to be defined properly and the idea was to model this in the same way a doctor would define the onset of a viral infection looking at symptoms present. The Reviewer is correct in the sense that, with more data and more input, this definition of onset might be refined by data-driven rules in the future.
